# Taxonomy of the genus *Epsilon* from China, with a new species and an updated key to the Oriental species (Hymenoptera, Vespidae, Eumeninae)

**DOI:** 10.3897/zookeys.910.35846

**Published:** 2020-02-10

**Authors:** Xue Zhang, Bin Chen, Ting-Jing Li

**Affiliations:** 1 Chongqing Key Laboratory of Vector Insects, Chongqing Key Laboratory of Animal Biology, Institute of Entomology and Molecular Biology, Chongqing Normal University, Chongqing 401331, China Chongqing Normal University Chongqing China

**Keywords:** *Epsilon dyscherum*, *Epsilon fujianense*, *Epsilon similimanasicum*, new combination, *Pararrhynchium subfistulosum*

## Abstract

A new species of potter wasps, *Epsilon similimanasicum***sp. nov.** is described and illustrated from Zhejiang, China. The holotype specimen of *Epsilon subfistulosum* (Wickwar, 1908) was examined and based on the characters of the type specimen, it is transferred from the genus *Epsilon* de Saussure to the genus *Pararrhynchium* de Saussure, as *Pararrhynchium subfistulosum***comb. nov.** In addition, an updated key to the Oriental species of *Epsilon* is provided.

## Introduction

The genus *Epsilon* de Saussure, 1855 includes 17 known species and one subspecies, among which 14 species and one subspecies are distributed in the Oriental region and three species in the Australian region. Giordani Soika (1994) comprehensively reviewed the genus with the conclusion that it contains nine species and one subspecies, and provided a good basis for further taxonomic study of the genus which lead to more publications (Gusenleitner 1996, 2011; Girish Kumar et al. 2014; and Selis 2017). To date, two species, *E. fujianense* Lee, 1981 and *E. dyscherum* (de Saussure) have been recorded from China (Lee 1981; Li et al. 2019). Except for these two species, one additional species from Zhejiang, China is recognized to be new to science, and is described and illustrated in detail. Meanwhile, because the existing descriptions and figures of the original description of *Odynerus subfistulosum* Wickwar, 1908 and in Giordani Soika (1941) appear to be inconsistent with the diagnosis of the genus *Epsilon*, such as the dentiform superior carina of the propodeum, and the sub-square tergum I which is basally truncated, slightly wider than long, and its lateral margins parallel, we examined the holotype specimen (NHMUK, type no. 18.314) deposited in the Natural History Museum, London. The study of the holotype of *O. subfistulosum* revealed that all the characters match the characters of the genus *Pararrhynchium* de Saussure. Further related characters and label information of the type specimen are given in this paper. Therefore, we suggest *Epsilon subfistulosum* (Wickwar, 1908) should be removed from the genus *Epsilon* to the genus *Pararrhynchium* de Saussure. Based on references and specimens, a key to the Oriental species is updated.

## Materials and methods

The specimens examined are deposited in Chongqing Normal University, Chongqing, China (CN; **CQNU**), the Natural History Museum, London, Great Britain (UK; **NHM**), American Museum of Natural History, New York, the United States (USA; **AMNH**), and Yunnan Agricultural University, Kunming, China (CN; **YNAU**). Descriptions were made under a stereomicroscope (Olympus SZ2-ILST). Measurements were taken as the maximal length of body parts under an image analyzer, all figures were taken with a stereomicroscope (KEYENCE-VHX-5000) attached to a computer in Chongqing Normal University, and the plates were arranged with Photoshop CS 6. Body length was measured from the anterior margin of head to the posterior margin of metasomal tergum II. Terminology follows Yamane (1990).

## Taxonomy

### Genus Epsilon

Taxon classificationAnimaliaHymenopteraVespidae

de Saussure, 1855

7E05E7D3-8A6D-5A78-9D1A-062A36984364

Epsilon de Saussure 1855: 229, 252; Giordani Soika 1994: 270–285; Girish Kumar et al. 2014: 5380–5385.

#### Type species.

*Odynerus dyscherus* de Saussure, 1853, by subsequent designation of van der Vecht, 1967: 31.

#### Diagnosis.

Clypeus much wider than long, with sparse or dense punctures (Figs 2, 13); cephalic fovea of female with two contiguous, deep pits (Figs 3, 12); tegula with broad lobe posteriorly, almost equal to parategula (Figs 9, 17); metanotum (Figs 7, 21) narrow and very protruding, with a vertical posterior face, flat or gently convex, and a horizontal dorsal face; propodeum (Figs 7, 21) short, without superior carina and with weak lateral carina; second submarginal cell with second recurrent vein nearly or completely interstitial with third submarginal cell (Figs 5, 14); tergum I without transverse carina basally, very short, ca. 2× as wide as long, and slightly narrower than tergum II (Fig. 20); tergum II (Figs 6, 20) usually with apical lamella (Giordani Soika 1994; Girish Kumar et al. 2014).

#### Distribution.

Oriental and Australian regions.

### Epsilon similimanasicum sp. nov.

Taxon classificationAnimaliaHymenopteraVespidae

55BC144F-AC37-526A-BEA0-3C60E649C768

http://zoobank.org/1DFFC638-2E66-492C-A6B1-68B0D0FB96EB

Figures 1–9

#### Material examined.

Holotype, ♀, China, Zhejiang Prov., Lin An City, Qingliang Mountain, Tianchi, 30°06'44"N, 118°56'26"E, 571 m, 3.VI.2012, Rui Zhang leg. (YNAU).

#### Description.

**Figures 1–9.**
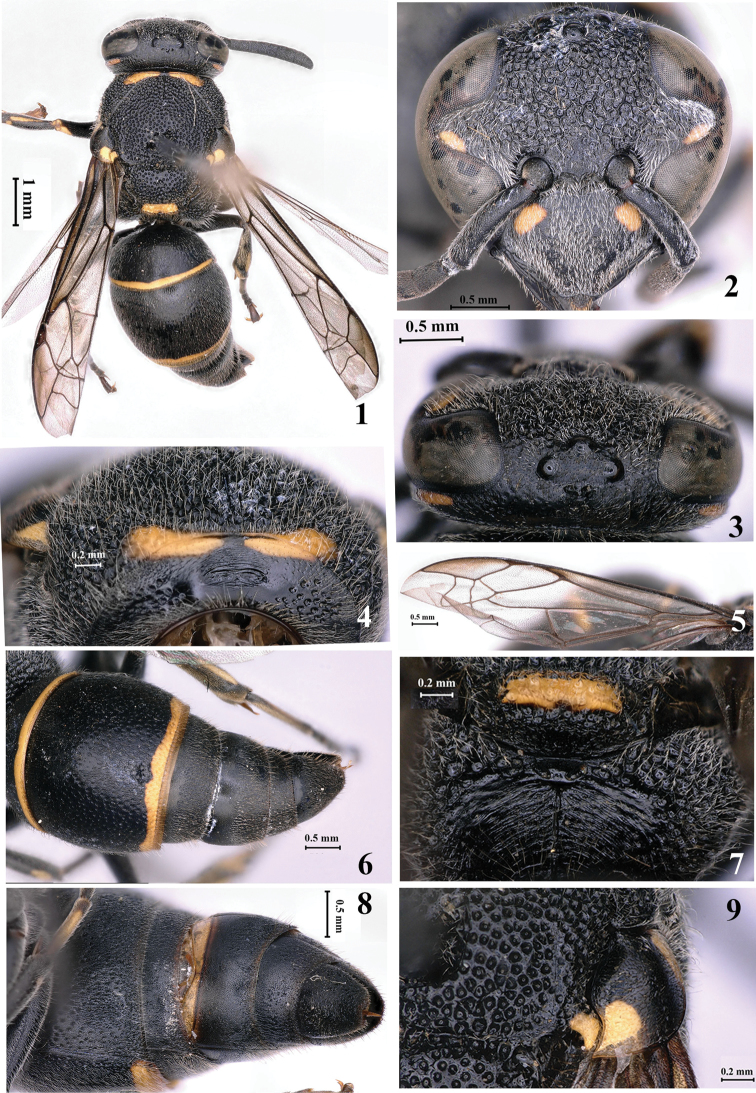
Female of *Epsilon similimanasicum* sp. nov. **1** Habitus (dorsal view) **2** clypeus **3** head (dorsal view) **4** anterior face of pronotum **5** wings **6** metasoma (dorsal view) **7** metanotum and propodeum **8** metasoma (ventral view) **9** tegula.

**Female** (Fig. 1): body length 7.5 mm. Black, with the following parts yellow: two lateral spots at the base of clypeus (Fig. 2), a small spot at the lower margin of ocular sinus, an elongated band on temple, an anterior interrupted transverse band on pronotum (Fig. 4), two spots on both anterior and posterior lobes of tegula, parategula, and horizontal dorsal face of metanotum (Fig. 7), a small apical spot on fore femur, small spots at outer sides of all tibiae, all tarsi ventrally, and apical bands of both terga I and II (Fig. 6). Body with white setae.

***Head.*** In frontal view, head 1.2× as wide as long; clypeus 1.4× as wide as long in front view, slightly emarginated at the middle of apex, with minute and sparse punctures, interspaces between punctures longer than diameters (Fig. 2); interantennal space convex, with a hump-like transverse carina connecting longitudinal carina at its middle (Fig. 2); frons coarsely punctate and reticulate; ocelli normal, diameter more than the distance between anterior and posterior ocelli, distance between posterior ocelli 1.6× as long as that between anterior and posterior ocelli (Fig. 3); interocellar area with micro-punctures; occipital carina developed laterally and weak dorsally (Fig. 3); cephalic fovea well developed and with two pits, total width of two pits distinctly shorter than distance between posterior ocelli (Fig. 3); punctures of vertex sparse, interspaces between punctures longer than diameters.

***Mesosoma.*** Mesosoma with punctures coarser than those on head; anterior vertical face of pronotum medially with fine and transverse striae and two connected elliptical pits at the lower part, and coarsely punctate on sides, pronotal carina complete, dorsal face with coarse and dense punctures, interspaces between punctures less than diameters (Fig. 4); mesoscutum with dense punctures, punctures just sparser than those of pronotum; mesepisternum with reticulate punctures; tegula with broad posterior lobe (Fig. 9); scutellum coarsely punctate medially and denser anteriorly and posteriorly; metanotum without tubercles, with horizontal dorsal face and vertical posterior face, the latter weakly convex in lateral view; propodeum short, both dorsal and lateral faces with coarse and reticulate punctures, posterior face almost vertical, broadly and shallowly depressed, and with distinct transverse striae and a longitudinal median carina (Fig. 7), lateral surface largely striate; second recurrent vein of fore wing separated from first recurrent one, and arched in the middle (Fig. 5).

***Metasoma.*** Tergum I short, 1.9× as wide as long in dorsal view, slightly narrower than (0.9×) tergum II, both terga I and II with sparse punctures, interspaces more than diameters; tergum II with apical lamella (Fig. 6); sternum II more or less medially depressed at the base; terga and sterna III–VI coriaceous (Figs 6, 8).

**Male.** Unknown.

#### Remarks.

This species is similar to *E. manasicum* Girish Kumar & Carpenter, 2014, but it can be distinguished from the latter and other species of the genus by the following characters: total width of two pits on the vertex distinctly less than distance between posterior ocelli (Fig. 3), anterior vertical face of pronotum with fine and transverse striae and two connected elliptical pits medially (Fig. 4), second recurrent vein of second submarginal cell separated from first recurrent one and arched in the middle (second recurrent vein of fore wing almost connected with first recurrent vein and rather arched in *E. manasicum*) (Fig. 5), and posterior face of propodeum with distinct transverse striae (Fig. 7).

#### Distribution.

China (Zhejiang).

#### Etymology.

The specific name *similimanasicum* is named after the similar species *E. manasicum*, combined with the Latin word *similis* (= similar).

### Epsilon fujianense

Taxon classificationAnimaliaHymenopteraVespidae

Lee, 1981

541755A1-3E49-572D-8923-E5095AC281AD

Figures 10–21

Epsilon fujianense Lee 1981: 198; Yeh and Lu 2007: 83–90; Tan et al. 2018: 109–149.

#### Material examined.

1♂, China, Guizhou Prov., Kaili Mountain, Fangxiang Town, Fangxiang Village, 26°26'22"N, 108°16'45"E, 890 m, 24.VI.2015, Zhenxia Ma & Pan Huang leg.; 1♂, China, Fujian Prov., Wuyishan City, Yangzhuang Town, East Village, 27°49'33"N, 117°58'56"E, 278 m, 26.VI.2014, Tingjing Li leg.; 1♂, China, Jiangxi Prov., Xinganshan, 29°7'38"N, 117°54'31"E, 223 m, 21.VI.2013, Michael Staab leg.; 1♂, China, Zhejiang Prov., Gutianshan, 29°12'53"N, 118°8'5"E, 366 m, 19.IV.2013, Michael Staab leg.; 1♀1♂, China, Guangxi Prov., Cenxi City, Malu Town, Lingyao Village, 22°52'58"N, 110°48'10"E, 69 m, 10.VI.2016, Zhenxia Ma leg.; 1♂, China, Jiangxi Prov., Xinganshan, 29°7'28"N, 117°54'36"E, 246 m, 6.VI.2013, Michael Staab leg.; 1♂, China, Jiangxi Prov., Xinganshan, 27°49'13"N, 117°55'46"E, 86 m, 19.IX.2015, Michael Staab leg.; 1♂, China, Chongqing Prov., Youyang County, Banqiao Town, Shuangqiao Village, 28°52'38"N, 108°49'25"E, 804 m, 13.VII.2012, Cheng Yang leg.; 2♂♂, China, Jiangxi Prov., Xinganshan, 27°49'13"N, 117°55'46"E, 86 m, 3.VII.2015, Felix Fornoff leg.; 1♂, China, Guizhou Prov., Tongren City, Jiangkou County, Heiwan Village, 27°50'39"N, 108°46'29"E, 536 m, 28.VI.2015, Zhenxia Ma leg.; 1♂, China, Jiangxi Prov., Xinganshan, 27°49'13"N, 117°55'46"E, 86 m, 14.VII.2015, Felix Fornoff leg.; 1♂, China, Jiangxi Prov., Xinganshan, 27°49'13"N, 117°55'46"E, 86 m, 13.VII.2015, Felix Fornoff leg.; 1♀, China, Guizhou Prov., Jiangkou County, 27°41'8"N, 108°49'44"E, 437 m, 1.XI.2018, Zhenkun Hu leg.; 1♀, China, Jiangxi Prov., Xinganshan, 27°49'13"N, 117°55'46"E, 86 m, 11.VII.2015, Felix Fornoff leg.; 1♀, China, Zhejiang Prov., Gutianshan, 29°12'53"N, 118°8'5"E, 366 m, 4.V.2013, Michael Staab leg.; 1♀, China, Anhui Prov., Liuanhuoshan County, Yuer Town, 31°13'53"N, 116°15'45"E, 98.4 m, 26.VII.2016, Yan Peng leg.; 2♀♀, China, Jiangxi Prov., Xinganshan, 27°49'13"N, 117°55'46"E, 86 m, 3.VI.2015, Felix Fornoff leg.; 1♀, China, Guangdong Prov., Lianxian County, Dongshan Mountain, 7.IX.1992, Jing Wang leg.; 1♀, China, Zhejiang Prov., Gutianshan, 29°14'33"N, 118°8'59"E, 309 m, 16.VII.2012, Michael Staab leg.; 1♂, China, Jiangxi Prov., Xinganshan, 27°49'13"N, 117°55'46"E, 86 m, 29.VI.2015, Felix Fornoff leg.

#### Diagnosis.

Clypeus in female wholly black, in male almost yellow except for apical margin (Figs 13, 15); cephalic fovea well developed, with two pits; total width of two pits distinctly shorter than distance between posterior ocelli (Fig. 12); anterior vertical face of pronotum medially with two separated elliptical depressions and indistinct fine transverse striae, but laterally almost smooth (Fig. 16); tegula with broad posterior lobe equal to parategula (Fig. 17); metanotum indistinctly faintly bi-dentate, lower vertical surface of metanotum coriaceous (Fig. 21); second recurrent vein of second submarginal cell close to first recurrent one, and nearly straight (Fig. 14); posterior surface of propodeum with few fine transverse striae and a median longitudinal carina (Fig. 21); tergum II with small or medium punctures, distances between punctures greater than diameters, and with short apical lamella (Fig. 20).

#### Distribution.

China (Zhejiang, Anhui, Jiangxi, Guangdong, Guangxi, Chongqing, Guizhou).

**Figures 10–21.**
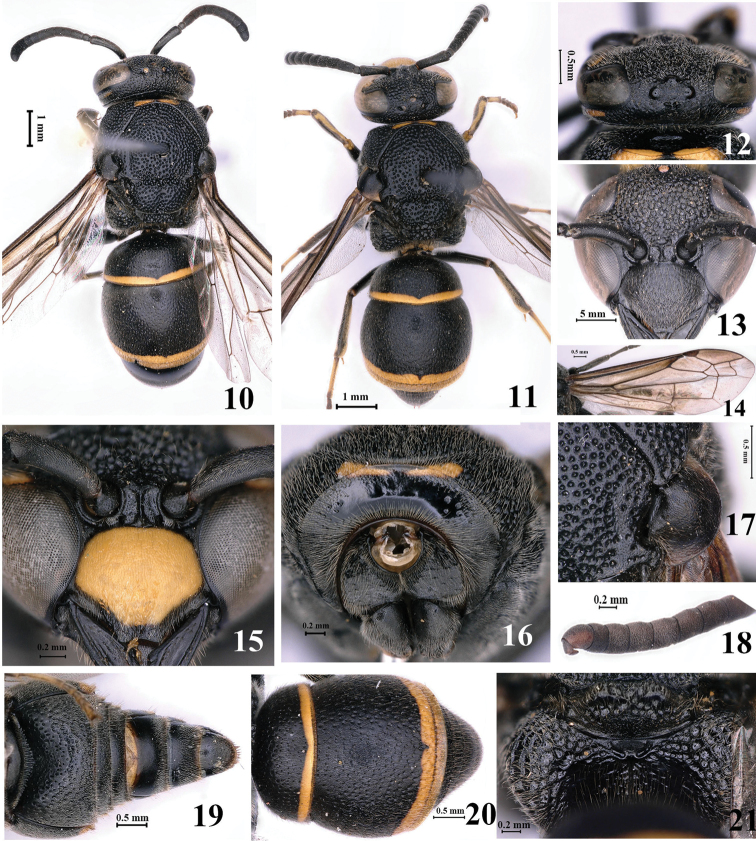
*Epsilon fujianense* Lee, 1981 **10, 12–14, 17, 21** female **11, 15, 16, 18–20** male **10** habitus (dorsal view) **11** habitus (dorsal view) **12** head (dorsal view) **13** clypeus **14** wings **15** clypeus **16** anterior face of pronotum **17** tegula **18** antennal flagellomeres II–XI **19** metasoma (ventral view) **20** metasoma (dorsal view) **21** metanotum and propodeum.

### Epsilon dyscherum

Taxon classificationAnimaliaHymenopteraVespidae

(Saussure, 1852)

6A213B10-B2E2-50DE-A8B3-C3EF9577DC66

Odynerus dyscherus de Saussure 1853: 175.
Odynerus xanthozonatus Williams 1928: 80, 82, 99; van der Vecht 1937: 290.
Epsilon dyscherus Gusenleitner 1996: 42; Li et al. 2019: 145, 147.

#### Material examined.

1♀, China, Hong Kong, Ping Shan Chai, hand net, 30.v.2015, UTM: 50Q KK 106883, 140 m, ref.: 0546.B.Hy.2, Christophe Barthélémy, leg.

#### Diagnosis.

Clypeus almost black, shallowly emarginated at apex, apical width about 1/3 of clypeal width; ocelli small, much less than distance between anterior and posterior ocelli; interocellar area with dense coarse punctures in female; parategula board basally; tergum II with punctures, interspaces between punctures generally greater than diameters, and with short apical lamella; figures as figs 68–70 in Li et al (2019).

#### Distribution.

China (Hong Kong); Philippines.

### Pararrhynchium subfistulosum

Taxon classificationAnimaliaHymenopteraVespidae

(Wickwar, 1908) comb. nov.

7E5058FD-EB59-5A0E-B05A-C25F09EB70DA

Figures 22–27

Odynerus subfistulosum Wickwar 1908: 118, 120;
Odynerus (Stenodyneroides) subfistulosum Wickwar 1908: Giordani Soika 1941: 249 (notes on female type).
Epsilon subfistulosum : Girish Kumar et al. 2014: 5384.

#### Material examined.

1♀, Sri Lanka (Ceylon), deposited in London, type no. 18. 314 (NHM).

#### Remarks.

**Figures 22–27.**
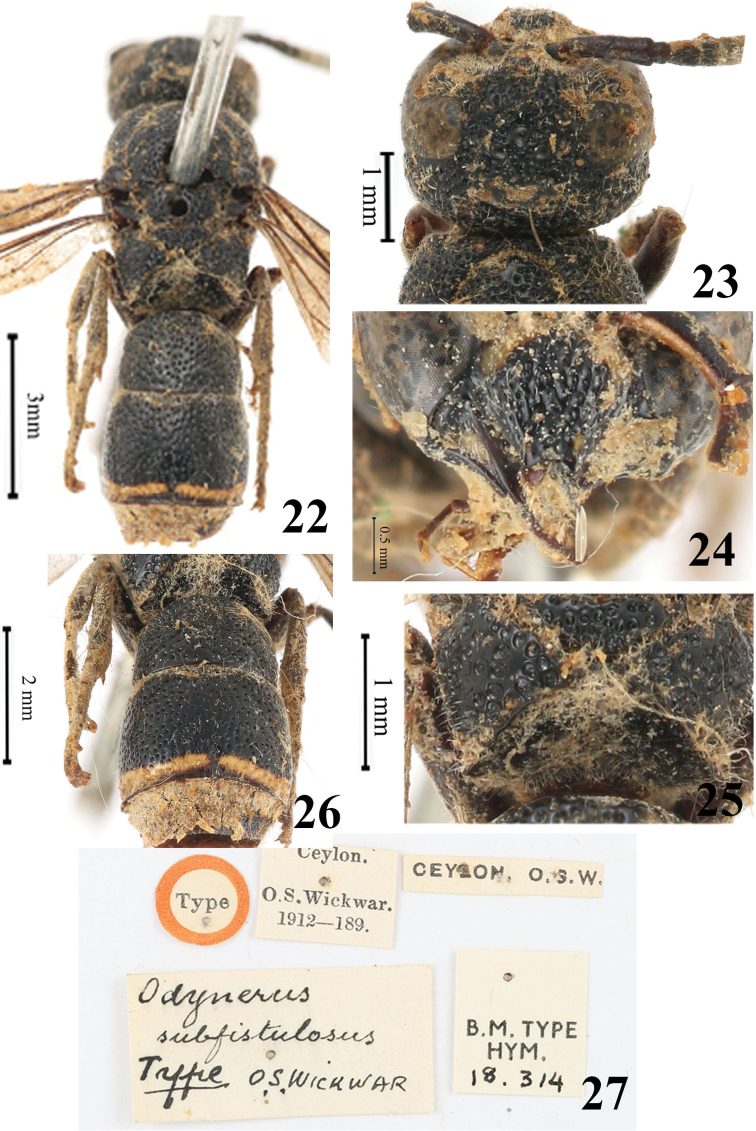
*Pararrhynchium subfistulosum* (Wickwar 1908), comb. nov. **22** habitus (dorsal view) **23** head (dorsal view) **24** clypeus **25** metasoma (dorsal view) **26** metanotum and propodeum **27** specimen information.

Wickwar (1908) described this specimen as belonging to the genus *Odynerus* Latreille. Having examined the same specimen, Giordani Soika (1941) placed *Odynerus subfistulosus* in the subgenus Stenodyneroides and then added the following characters: superior carina of propodeum dentiform, tergum I sub-square, basally truncated, slightly wider than long, and its lateral margins parallel, tergum II very slightly longer and slightly wider than tergum I and strongly reflected at the apex. With those characters in mind, we examined the holotype specimen and found that more characters match to those of the genus *Pararrhynchium*, such as one large cephalic fovea on the vertex (Fig. 23), the clypeus coarsely punctate and more or less emarginated at apex (Fig. 24), the scutellum and mesoscutum ﬂattened (Fig. 22), the propodeum with a superior carina (Fig. 26), and tergum I with transverse carina basally (Figs 25, 26). Therefore, we suggest that *Odynerus subfistulosum* Wickwar, 1908 should be transferred to the genus *Pararrhynchium*.

#### Distribution.

Sri Lanka.

### Key to the Oriental species of *Epsilon* (modified from Giordani Soika 1994)

**Table:** 

1	Tergum II with large and dense punctures, interspaces between punctures equal to or less than diameters	**2**
–	Tergum II with small or medium and sparse punctures, interspaces between punctures generally greater than diameters	**3**
2	Punctures on tergum II large and dense, similar to or greater than those of pronotum; posterior lobe of tegula narrow	***E. burmanicum* (Bingham, 1897)**
–	Punctures on tergum II smaller than those of pronotum; posterior lobe of tegula broad	***E. chikmagalurense* (Lambert, 2008)**
3	Clypeus very deeply emarginated at the apex, forming two wide and blunt teeth (Giordani Soika 1994: fig. 63)	***E. vechti* Giordani Soika, 1994**
–	Clypeus different from above	**4**
4	Ocelli smaller, much less than distance between anterior and posterior ocelli	**5**
–	Ocelli normal, approximately the same size as distance between anterior and posterior ocelli	**6**
5	Interocellar area with dense coarse punctures; without transverse carina above anterior ocellus; gena with very dense punctures, interspaces less than diameters	***E. dyscherum* (de Saussure, 1853)**
–	Interocellar area with sparse coarse punctures; with transverse carina above anterior ocellus; gena with sparse punctures, interspaces more than diameters	***E. grandipunctatum* Gusenleitner, 1996**
6	Tergum II with long apical lamella	**7**
–	Tergum II with short or without apical lamella	**9**
7	Body with red spots and bands (Gusenleitner 2011: fig. 9); clypeus with coarser punctures (Gusenleitner 2011: fig. 10)	***E. rubromaculatum* Gusenleitner, 2011**
–	Body with yellow spots and bands; clypeus with fine punctures (Giordani Soika 1994: fig. 61)	**8 *E. manifestum* (Smith, 1858)**
8	Base of clypeus with two yellow spots laterally (Giordani Soika 1994: fig. 61)	***E. m. manifestum* (Smith, 1858)**
–	Base of clypeus with yellow band	***E. m. crassipunctatum* Gusenleitner, 1991**
9	Tergum II without lamella	**10**
–	Tergum II with short lamella	**11**
10	Clypeus with coarse dense punctures, interspaces less than diameters (Giordani Soika 1994: fig. 62); cephalic fovea large with two deep pits; legs with yellow spots	***E. laboriosum* (Smith, 1864)**
–	Clypeus with small sparse punctures, interspaces more than diameters (Giordani Soika 1994: fig. 60); cephalic fovea small and with two shallow pits; legs entirely black	***E. tinctipenne* (Walker, 1860)**
11	Clypeus deeply emarginated at the middle of apex (Selis 2017: fig. 2)	***E. rufipes* Selis, 2017**
–	Clypeus slightly emarginated at the middle of apex (Figs 2, 13)	**12**
12	Anterior face of pronotum with few sparse punctures on sides (Fig. 16); metanotum with two blunt teeth (Fig. 21)	**13**
–	Anterior face of pronotum with distinct coarse and dense punctures on sides, interspaces less than diameters (Fig. 4); metanotum without tubercles, with flat horizontal dorsal face	**14**
13	Clypeus black in female (Fig. 13) and almost yellow except for apical margin in male (Fig. 15); clypeal surface obscurely areolate-rugulose	***E. fujianense* Lee, 1981**
–	Clypeus with two lateral yellow spots basally in female, and a broad curved interrupted yellow band along the basal margin in male; clypeal surface not distinctly punctate	***E. achterbergi* Giordani Soika, 1994**
14	Total width of cephalic fovea pits nearly equal to distance between posterior ocelli; anterior surface of pronotum with dense transverse striae medially; second recurrent vein of second submarginal cell almost connected with first recurrent one, and wholly arched; posterior face of propodeum with a few fine transverse striae or almost smooth (Girish Kumar et al. 2014: figs 7, 8, 10, 11)	***E. manasicum* Girish Kumar & Carpenter, 2014**
–	Total width of cephalic fovea pits distinctly shorter than distance between posterior ocelli (Fig. 3); anterior surface of pronotum with two connected depressions and fine transverse striae medially (Fig. 4); second recurrent vein of second submarginal cell separated from first recurrent vein and arched at the middle (Fig. 5); posterior face of propodeum with distinct transverse striae and not smooth (Fig. 7)	***E. similimanasicum* sp. nov.**

## Supplementary Material

XML Treatment for Epsilon

XML Treatment for Epsilon similimanasicum

XML Treatment for Epsilon fujianense

XML Treatment for Epsilon dyscherum

XML Treatment for Pararrhynchium subfistulosum
